# Perceived invulnerability in traffic: illusion of control, desire for control, risk perception, and traffic-locus of control

**DOI:** 10.3389/fpubh.2025.1626481

**Published:** 2025-10-03

**Authors:** Elena-Cristina Nae, Camelia Truța, Ana-Maria Cazan

**Affiliations:** ^1^Romanian Academy, Institute of Philosophy and Psychology "Constantin Rădulescu-Motru", Bucharest, Romania; ^2^Faculty of Psychology and Education Sciences, Transilvania University of Brașov, Brașov, Romania

**Keywords:** risky driving behaviors, illusion of control, desire for control, risk perception, traffic-locus of control, perceived invulnerability

## Abstract

**Introduction:**

This study examined the psychological predictors of risky driving behavior, focusing on traffic-locus of control, illusion of control, desire for control, and risk perception—as key components of perceived invulnerability.

**Methods:**

Two main hypotheses were tested: the first (H1) included two sub-hypotheses, H1a, that perception of invulnerability predicts risky driving, and H1b, that this relationship is moderated by driving experience; the second hypothesis (H2), was that risk perception mediates the relationship between traffic-locus of control, driving experience, and risky driving behaviors. A sample of 115 drivers completed standardized self-report questionnaires.

**Results:**

Results partially supported the first hypothesis: perceived invulnerability, operationalized through illusion of control, desire for control, and risk perception predict risky driving behavior. However, driving experience did not moderate any of the observed relationships. The second hypothesis was also partially confirmed. A two-step mediation model revealed that traffic-locus of control and driving experience predicted risk perception, while the full model explained 47% of the variance in risky behavior. Risk perception partially mediated the relationship between beliefs about other drivers and risky driving. Additionally, beliefs in fate and luck were directly associated with lower risk engagement. Contrary to expectations, driving experience did not moderate any of the key relationships, suggesting that these psychological patterns remain influential regardless of experience level.

**Discussion:**

These findings underscore the complex interplay between control beliefs and perceived risk in driving contexts, offering insights for interventions targeting cognitive distortions and overconfidence that contribute to hazardous driving practices.

## 1 Introduction

Road safety remains a major global concern, including in Romania, where efforts to improve it often fall short despite official commitments. According to the World Health Organization (1), approximately 1.19 million people die in road traffic accidents each year, and 20–50 million suffer serious injuries. At the European Union level, over 20,000 fatalities were reported in 2022 (2) with Romania (81 deaths per million inhabitants) and Bulgaria (82) ranking highest, well above the EU average of 46 (2). These statistics highlight the urgent need to improve road safety not only through infrastructure and education, but also by addressing the psychological factors that drive risk-taking behavior. Risky behavior behind the wheel significantly increases the risk of traffic accidents (1). In Romania, risky driving behaviors such as speeding, alcohol consumption, and distracted driving are widely recognized as contributing factors to severe road accidents, although detailed national statistics quantifying their exact impact remain limited in publicly accessible reports. Nonetheless, European-level analyses show that these behaviors are determinants of road traffic accidents, particularly in countries with high mortality rates like Romania (2). This highlights the importance of understanding the psychological mechanisms that drive such risk-taking behaviors among Romanian drivers.

One psychological factor that warrants critical attention is perceived invulnerability, a cognitive bias in which individuals underestimate personal risk (3, 4). Many drivers believe they are safer than others, despite engaging in dangerous behaviors. This overconfidence may underlie a significant proportion of preventable road accidents. Key dimensions of this perceived invulnerability include the illusion of control (5, 6), desire for control (7), and risk perception (8).

The *illusion of control* is a psychological phenomenon in which individuals believe they have more control than they do over their own behavior or their surrounding environment (72). In the context of road transport, the illusion of control describes drivers’ tendency to overestimate their ability to manage risky situations and to underestimate real dangers. For example, a driver might think, “If something happens, I’m skilled enough to avoid an accident,” which leads to a misjudgement of their true level of control. In Langer’s [(5), p. 311] words, the illusion of control is defined as “an expectation of a personally improbable probability of success greater than would be warranted by objective analysis.” Drivers might feel they have a higher chance of success if they personally manage a situation than if another person controls the situation, but their belief in personal control might be completely illusory.

In contrast, the *desire for control* refers to a motivational trait that reflects an individual’s general preference to be in control of situations and outcomes (7). Unlike the illusion of control, which is a cognitive bias or distortion, desire for control is a stable psychological tendency that influences how people approach decision-making and risk. In driving, individuals with a high desire for control may engage in behaviors aimed at maintaining or asserting control, which can interact with their perceptions of risk and vulnerability.

Risk perception involves subjective judgments about the likelihood of getting harm (8). Prior research has shown that cognitive biases, such as illusion of control and low risk perception, contribute to risky driving behavior. Drivers with a heightened illusion of control tend to overestimate their abilities and underestimate the likelihood of accidents, which has been linked to speeding and aggressive driving (9, 10). Similarly, low risk perception correlates with traffic violations and a reduced propensity to follow safety regulations (3, 11). Also, these factors may lead to distorted driving judgments, thereby contributing to risky behavior such as dismissing warnings or neglecting traffic regulations (9, 57).

The locus of control (LoC), a concept developed by Julian Rotter within social learning theory, describes how individuals perceive the relationship between their actions and outcomes (12, 73). It distinguishes between two types: internal locus of control and external locus of control. Individuals with an internal locus of control believe that personal characteristics, efforts, and behaviors directly influence life events. They are typically more motivated, proactive, and take responsibility for their actions, which leads to positive outcomes such as goal achievement and well-being (13, 14). In contrast, individuals with an external locus of control attribute outcomes to external forces such as luck, fate, or other people. This belief can reduce their initiative, increase feelings of helplessness, and is often associated with higher levels of anxiety and stress (15).

This general framework has been adapted to road traffic behavior through the concept of Traffic-Locus of Control (T-LoC) (6). T-LoC assesses how drivers explain causes of traffic events such as accidents or risky situations. Drivers with an internal traffic-locus of control tend to believe that their driving skills and behavior influence outcomes. In contrast, those with an external traffic-locus of control attribute traffic events to external factors like road conditions, other drivers, or luck. Integrating perceived invulnerability with T-LoC may help explain why some drivers consistently underestimate personal risk while also denying responsibility for dangerous outcomes.

Individual differences in risk perceptions and risky driving behaviors are determined by factors such as age and driving experience. Young and inexperienced drivers are particularly vulnerable to road crashes. The highest crash risk is recorded during the first 6 months of independent driving (16), when drivers are more likely to misjudge traffic situations, succumb to fatigue, especially at night, and be influenced by peer passengers, increasing the likelihood of risky decisions (17).

Romanian national statistics confirm this trend: in 2023, one in five drivers who died in road crashes were aged 18–30, and 58.7% of all young road user fatalities were vehicle drivers (18). These data emphasize the critical role of driving experience as a predictor of crash risk, beyond chronological age.

Less experienced drivers might have worse hazard perception skills, be more prone to cognitive biases like the illusion of control and have a lower understanding of risk (8, 17). These issues can make them more likely to engage in risky driving behaviors. This study includes driving experience as a possible moderator to see if it affects the links between psychological factors and risky driving behavior.

So far, the specific interaction between perceived invulnerability and how drivers attribute outcomes, whether to their own actions or to external circumstances (19), has not been thoroughly investigated, especially in countries with elevated traffic fatality rates, such as Romania.

The current study addresses existing gap by exploring how the key dimensions of perceived invulnerability (illusion of control, desire for control, and risk perception) relate to risky driving, and whether risk perception mediates the influence of traffic-locus of control on risk-taking. Furthermore, we investigate whether risk perception mediates the influence of traffic-locus of control on risk-taking, and whether driving experience moderates these relationships given the limited prior research examining these combined psychological factors in Romania, the present study adopts an exploratory approach to identify potential psychological mechanisms contributing to risky driving. Choosing Romanian drivers as the sample aligns with the study’s aims, given Romania’s persistently high road fatality rate (2). This context offers a unique opportunity to investigate the psychological mechanisms underlying risk-taking behavior in one of the EU’s most traffic-vulnerable populations. This context offers a unique opportunity to generate preliminary insights and inform future, more focused research and prevention efforts targeting cognitive distortions in traffic contexts.

## 2 Literature review

### 2.1 Risk and risk-taking behaviors in traffic

Hazardous driving behaviors are significant determinants of serious road accidents. Risky actions such as speeding (20, 74), driving under the influence (74), failure to use seat belts (20), and disregarding traffic signals (21) are major contributors to serious and fatal road accidents.

This pattern is also evident in Romania. In 2023, the Romanian Police (22) reported that inappropriate speeding led to 733 serious accidents, resulting in 312 fatalities and 607 severe injuries. Additionally, 8,368 drivers were sanctioned for driving under the influence of alcohol, out of a total of 53,961 road-related criminal offenses (23). Furthermore, in the first 7 months of 2024, IGPR (24) data revealed that 63.6% of all serious accidents were caused by speeding, failure to yield, and driving under the influence of alcohol or drugs. Over 6,000 driving licenses were suspended for alcohol-related offenses during this period (25). Broader analyses from 2023 showed that rule violations such as speeding and other forms of non-compliance with traffic laws accounted for approximately half of all serious accidents (23).

Risky driving remains a primary cause of traffic accidents in Romania. According to the latest data from the Romanian Police (24) drivers were responsible, entirely or partially, for 58% of serious accidents. Common risky behaviors include excessive speeding, failure to yield, aggressive maneuvres, and disregard for traffic signals. Notably, inappropriate speed remains one of the leading causes of fatal crashes, as highlighted in the Romanian Road Safety Bulletin 2023 (24).

Although somewhat dated, previous research has consistently shown that not wearing seat belts increases the risk of fatal injury by approximately 40% (26, 27), while failure to comply with traffic signals accounts for about 7% of fatal accidents. These findings are supported by European data (28), which place Romania among the highest in the EU for seat-belt violations (24 infringements per 1,000 inhabitants during RoadPol checks). Moreover, 84% of rear-seat passengers admit to frequently traveling without wearing a belt. Aggressive behaviors such as unsafe lane changes and tailgating further contribute significantly to accidents (26, 29). Recent international reports confirm that these behaviors remain critical road safety concerns (20, 21).

In this study, we measured three key dimensions of risky driving behaviors: attitudes toward rule-breaking and speeding, attitudes toward reckless driving by others, and attitudes toward alcohol use and driving. These dimensions are widely recognized as major contributors to serious road accidents both internationally (20, 24) and within Romania (24, 26). Their consistent identification as leading factors underscores their importance for understanding risky driving in the Romanian context.

### 2.2 Perceived invulnerability: a dangerous illusion in traffic

Two key cognitive biases have been identified as central to how individuals perceive their safety on the road: optimism bias [the tendency to believe that negative outcomes are less likely to happen to oneself; (30)] and the illusion of control. These distortions contribute to the broader phenomenon of perceived invulnerability, a psychological state in which drivers underestimate the risks associated with traffic situations. According to McKenna (4), both biases foster an overly positive outlook on one’s driving abilities and potential outcomes, generating a false sense of security—even in objectively hazardous conditions. These biases have been empirically linked to increased engagement in speeding, mobile phone use while driving, and other risky behaviors (10), being especially common among young or inexperienced drivers, who often possess limited hazard perception skills but elevated confidence in their driving ability (3, 75).

These constructs are among the most frequently examined in traffic psychology and are widely recognized for their influence on risky driving behaviors (10). Hăvârveanu (30) suggests that the illusion of control is linked to self-evaluation errors, with the optimism bias playing a role in generating this illusion. Though the mechanisms differ, these illusions are connected by their effects and likely correlate positively. Havârneanu (30) tested the illusion of invulnerability among 160 traffic participants by asking them to estimate the likelihood of being involved in various accidents, ranging from minor to fatal. The results showed that participants rated all types of accidents as unlikely, with no event exceeding a 50% chance. Most predicted a minor accident, and overall, participants saw themselves as vulnerable in fewer than one-third of cases.

Nevertheless, both contribute to the broader phenomenon of perceived invulnerability, which underpins many risky behaviors in traffic. In this study, however, we focus specifically on the illusion of control and the desire for control as psychological variables influencing drivers’ perception of risk and decision-making on the road.

McKenna (4) studied how the illusion of control influences risk perception in traffic. He distinguished it from the optimism bias, noting that while the latter arises from general positive expectations, the illusion of control occurs only when outcomes seem influenced by personal skills. For instance, the illusion of control applies to avoiding car accidents but not to events like earthquakes. In a study, McKenna (4) found that participants perceived the risk of accidents to be lower when driving than when being passengers, due to their stronger sense of control. The study demonstrated that perceived risk decreases with greater controllability, especially in active roles like driving. This highlights control as a key factor in drivers’ sense of invulnerability, which diminishes when control is absent (30).

Recent research has explored how drivers’ desire for control and illusion of control influence engagement in risky behaviors. Nees et al. (76) examined the relationships among various driving styles, desire for control, illusion of control, and self-reported risky driving behaviors. Their findings indicate that maladaptive driving styles, such as “risky” and “angry,” were negatively correlated with proactive aspects of desire for control and positively correlated with illusion of control. In other words, drivers who reported more aggressive or risk-prone behaviors tended not to exhibit a conscious desire to maintain control but were more likely to hold an inflated belief in their ability to manage hazardous situations. These results suggest that the illusion of control may be a more significant predictor of risky driving than the explicit motivation to exert control.

In a complementary study, Boua et al. (77) explored the relationships between control beliefs, risk perception, and traffic safety behaviors among Moroccan drivers. They found that higher perceived control over traffic risks was associated with lower risk perception and a greater tendency to engage in risky driving, supporting the illusion of control theory (5). This effect was stronger among young and inexperienced drivers. In contrast, higher risk perception was positively associated with safer driving behaviors. Additionally, older drivers tended to drive more safely than younger ones, even when their risk perception was low.

### 2.3 Traffic-locus of control

As previously defined, the concept of traffic-locus of control distinguishes between drivers with an internal T-LoC who believe their driving skills and behavior influence outcomes, and those with an external T-LoC who attribute events to external factors such as road conditions, other drivers, or luck. Research by Özkan and Lajunen (6), as well as by Salminen and Klen (31), showed that drivers with a higher internal T-LoC reported significantly fewer violations, errors, and accidents, whereas those with a more external T-LoC (e.g., attributing outcomes to fate or other drivers) displayed more risky behaviors and had a greater tendency to externalize responsibility. These findings emphasize the predictive value of T-LoC in road safety and support its use in understanding individual differences in driving behavior.

In the present study, we used the Romanian adaptation of the *Traffic Locus of Control Scale* developed by Măirean et al. (32), which is culturally validated and preserves the multidimensional structure of the original instrument of Özkan & Lajunen (6). This adaptation notably incorporates religiosity as an external control dimension, acknowledging the influence of fatalistic and divine control beliefs on driving behavior. Măirean et al. (32) found that Romanian drivers with a higher internal T-LoC demonstrated greater risk perception and engaged less in sensation seeking and risky driving behaviors. Conversely, those with a more external T-LoC exhibited riskier driving styles and committed more traffic offenses. However, the relationships between T-LoC and actual traffic outcomes like accidents were generally weak.

Religious beliefs can significantly shape driver behavior through fatalistic attitudes, which involve accepting events as predetermined. Such attitudes often lead to underestimating risks, neglecting safety measures like seat belt use, speeding, and dangerous maneuvers (33). Yet, in some cultures, religiosity encourages adherence to rules and lower risk-taking (34). International research supports the link between fatalistic beliefs as a form of divine control and higher risk perception as well as risky pedestrian behaviors (35, 36). Although these studies focus on pedestrians, the findings suggest that an external locus of control related to divine influence may similarly affect drivers’ risk perception and behavior. Thus, while LoC is a broad psychological trait, T-LoC is a context-specific construct that more accurately captures drivers’ perceived control in traffic situations. Understanding this distinction is essential for analyzing the psychological factors underlying road behavior.

### 2.4 Driving experience and risk misperception

Driving experience has emerged as a key predictor of safe driving, often outweighing age-related effects (37). Although age and experience are typically correlated, studies suggest that it is the lack of experience (particularly in the first years of driving) that most strongly predicts errors leading to accidents. Furthermore, driving experience interacts with other individual characteristics, such as gender. Men are more prone to adopt aggressive driving styles, underestimate risks, and overestimate their abilities (38), leading to a higher incidence of traffic violations and fatal crashes (39). Nevertheless, the 19–24 age group remains the most at-risk segment, regardless of gender, reinforcing the centrality of experience acquisition in shaping safe driving behavior (18).

Importantly, recent research suggests that experience does not necessarily eliminate cognitive distortions, such as the illusion of control, defined as the tendency to overestimate one’s ability to manage traffic situations. A Romanian study found that this bias positively predicts risky driving, with risk perception acting as a mediator: drivers who overestimate their control perceive less risk and consequently engage in more hazardous behavior (32). Moreover, a study by Navarro et al. (40) demonstrated that both novice and experienced drivers fall victim to the Dunning–Kruger effect: while novice drivers increased their confidence after simulated driving tasks, experienced drivers maintained high self-assessments regardless of actual performance. This suggests that overconfidence can persist even with increasing experience, potentially reinforcing risky behavior rather than correcting it.

Together, these findings highlight the complex role of driving experience in traffic safety. While experience contributes to improved skills and lower crash rates over time, it can also be accompanied by overconfidence and distorted risk appraisals, which may diminish the protective effects of experience unless explicitly addressed in driver education and behavioral interventions.

## 3 Study framework and hypotheses

This study proposes a comprehensive conceptual model for the understanding of the psychological underpinnings of risky driving behaviors, centered on the construct of perceived invulnerability in traffic. Our framework posits that perceived invulnerability is a multifaceted construct, operationalized through the interplay of illusion of control, desire for control, and risk perception. Complementing this, we introduce traffic-locus of control, encompassing its distinct subdimensions: destiny-luck, religious beliefs, other drivers, internality, and vehicles/environment, as a crucial antecedent influencing these perceptions. The study is based on the premise that perceived invulnerability leads to risk-taking and traffic rule violations.

Several theories aim to explain how drivers adjust their behavior in response to perceived risk. For example, risk compensation suggests that drivers adjust their behavior based on perceived risk, becoming more cautious in high-risk situations and more relaxed when they feel safe (41). However, this adjustment does not necessarily eliminate risky behavior, as drivers may deliberately increase their risk-taking once they perceive safety-enhancing measures or favorable conditions.

The Risk Homeostasis Theory goes further by proposing that drivers strive to maintain a stable, individually acceptable level of risk (42). This means that when perceived risk is low, drivers may intentionally engage in more hazardous behaviors (e.g., speeding, overtaking), whereas a heightened perception of risk may not reduce risky driving, but rather trigger compensatory strategies that sustain the desired “target level” of risk. In this sense, a higher risk perception can coexist with persistent or even elevated risky behaviors, as drivers calibrate their actions to maintain equilibrium.

The Zero-Risk Theory suggests that drivers tolerate risk up to a certain threshold; once perceived risk surpasses this boundary, compensatory mechanisms are activated to restore a sense of safety (43). Importantly, this implies that below that threshold, drivers may underestimate dangers and engage in behaviors that appear risky, precisely because they do not subjectively register the situation as threatening.

Finally, the Task-Capacity Model emphasizes the dynamic balance between driving demands and drivers’ perceived abilities (44). When drivers believe their skills or the road environment adequately match the demands, they may push limits and adopt riskier maneuvers. Conversely, if they feel their capacity is exceeded, they may adopt avoidance strategies, but this threshold is subjective and shaped by individual differences in risk perception.

However, understanding risk perception in traffic and its impact on driving behavior can be enhanced by considering cognitive processes and individual characteristics that influence how individuals perceive and assess risk and their ability to control it. Despite regulations and technological improvements in traffic systems, distorted risk perception can still lead to accidents (45).

**Figure 1:**
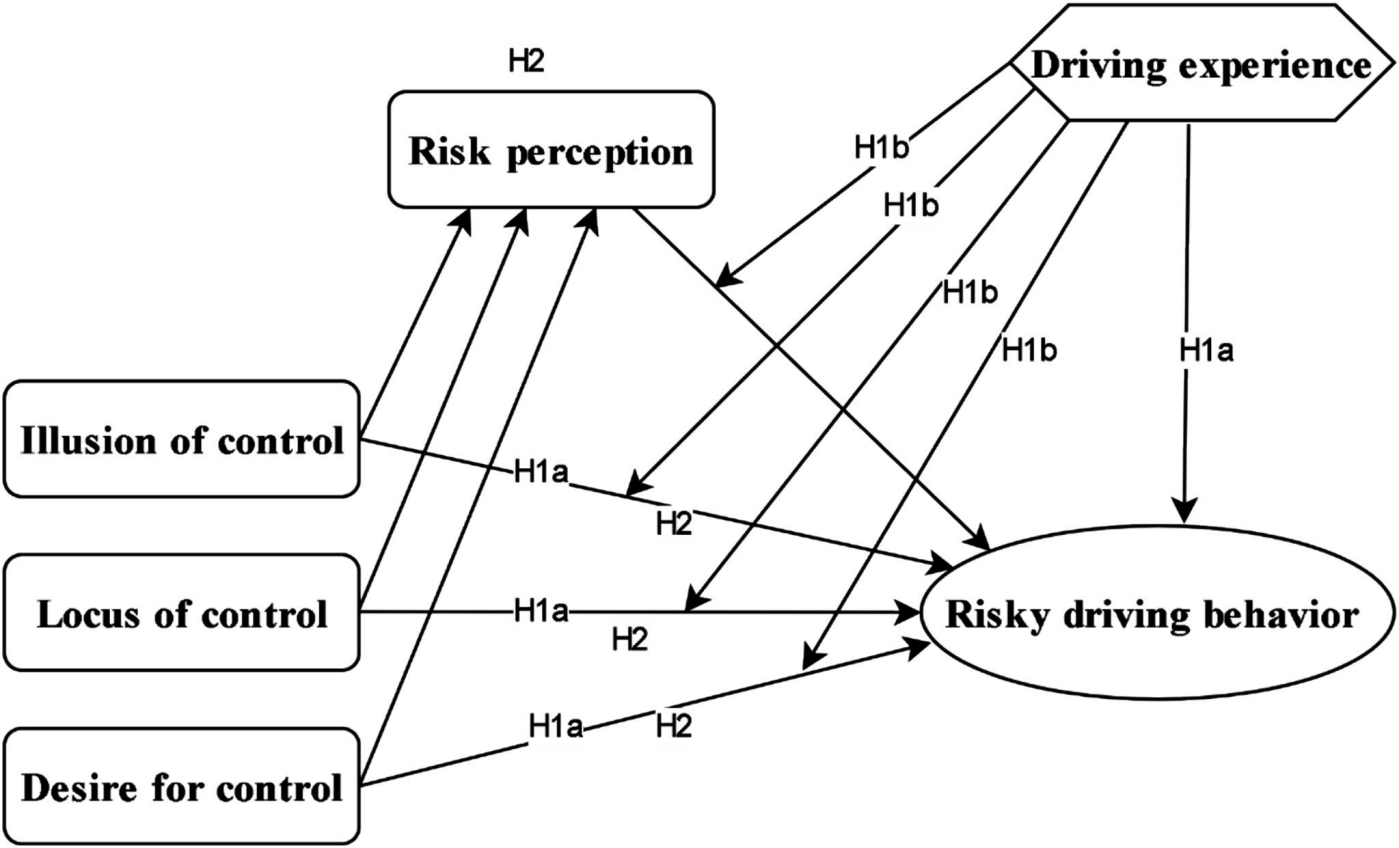
The proposed research model.

Building upon these foundational theories, our study presents a novel integration of cognitive-perceptual variables to explain individual differences in risky driving. Specifically, we propose the following hypotheses (Figure 1):

1. Perceived invulnerability, encompassing an individual’s illusion of control and desire for control, predicts risky driving behaviors (H1a). This relationship, however, is not uniform across all drivers; driving experience will significantly moderate the link between perceived invulnerability and risky driving, such that less experienced drivers may be more susceptible to the detrimental effects of invulnerability perceptions (H1b).
2. Risk perception mediates the relationship between traffic-locus of control (destiny-luck, religious beliefs, other drivers, internality, vehicles/environment), driving experience, and risky driving behaviors, even when accounting for moderation effects (H2).

By explicitly incorporating these cognitive-perceptual factors, illusion of control, desire for control, and a nuanced understanding of risk perception alongside traffic-locus of control, this framework extends existing traffic psychology models by delving into the subjective interpretations and personal beliefs that shape drivers’ subjective risk thresholds.

## 4 Methods

### 4.1 Participants

The sample consisted of individuals from diverse social backgrounds, age groups, and educational levels. The study was conducted on a convenience sample of young and adult drivers. The total number of participants was 115, aged between 19 and 74 years, with a mean age of 29.83 and a standard deviation of 13.05. Of these, 73 were female (63.5%) and 42 were male (36.5%).

Participants voluntarily joined the study after being informed about its purpose and giving their consent. To be eligible, participants needed to have held at least a category B driving license. Participants reported an average driving experience of 8.7 years (SD = 10.08). Exclusion criteria included refusal to sign the consent form, lack of a category B license, driving less than 100 kilometers in the past year, and incomplete survey responses. The questionnaire was administered online to all participants over a four-week period, distributed through social media platforms.

### 4.2 Measures

*Risky driving behavior* was measured using the *Self-Reported Risky Driving Behavior* (29). The questionnaire assesses the extent to which drivers’ styles include risky behaviors. Participants selected the statements that best reflected their attitudes in traffic situations. The scale comprises 16 items grouped into three factors: 1. *Attitudes toward rule-breaking and speeding* (11 items); 2. *Attitudes toward reckless driving of others* (3 items); *3. Attitudes toward alcohol use and driving* (2 items). Items were rated on a 5-point Likert scale (1 = Strongly Agree, 5 = Strongly Disagree), with several items reverse-scored. In the study conducted by Măirean et al. (32), the internal consistency (Cronbach’s Alpha) was 0.84, while in the current study it was 0.81.

The *Self-Reported Risky Driving Behavior* scale was selected due to its ability to capture multiple key dimensions of driving risk within a single instrument. Unlike other measures that focus on a single aspect of risk-taking—such as speeding, sensation-seeking, or aggression [e.g., the *Driving Anger Expression Inventory*—(46); the *Driving Behavior Questionnaire*—(47); or the *Sensation Seeking Scale*—(48)]—this scale offers a broader and more integrative assessment of risky driving, encompassing attitudes toward rule violations, alcohol use, and perceptions of others’ behaviors. Its multidimensional structure and demonstrated psychometric reliability, including in Romanian samples (32), make it particularly suitable for investigating risky driving behaviors in this context.

*Traffic-locus of control* was measured through the *Traffic-Locus of Control Scale* (32) adapted for the Romanian population. The scale consists of 50 items distributed across six subscales: 1. *Fate/Luck* (16 items); 2. *Religious Beliefs* (8 items); 3. *Desirability* (9 items); 4. *Other Drivers* (6 items); 5. *Internality* (5 items); 6. *Vehicle and Environment* (6 items). Responses were rated on a 5-point Likert scale (1 = Strongly Disagree, 5 = Strongly Agree). Internal consistency (Cronbach’s Alpha) ranged from 0.68 to 0.91 in the current study, which is comparable to the values reported by Măirean et al. (2016), who found Alpha coefficients ranging from 0.62 to 0.92.

*Illusion of control* was measured using the *Illusion of Control Scale* (49)—This unidimensional scale uses 10 scenarios to assess participants’ perceptions of control in risky traffic situations. Participants rated their control over each scenario, such as “Causing a serious accident while intoxicated.” Ratings used a 5-point Likert scale (1 = No Control, Luck-Based, 5 = Complete Control). Higher scores indicate stronger illusions of control. Cronbach’s Alpha was 0.73 in the current study, slightly higher than the value of 0.66 reported by Stephens and Ohtsuka (10).

*Desire for control* was measured using the *Desirability of Control Scale* (7)—This scale measures the desire for control in daily activities with 20 items, e.g., “I try to avoid situations where someone else tells me what to do.” Responses were rated on a 7-point Likert scale (1 = Does not apply at all, 7 = Always Applies). Higher scores indicate a stronger desire for control. Internal consistency was 0.75 in the current study, similar to the original findings by Burger and Cooper (7), who reported an Alpha of 0.80 and a test–retest reliability of 0.75.

*Risk perception* was measured through the *Risk Perception Questionnaire* (50)—This questionnaire evaluates perceived risk in traffic situations, with 34 items forming a single total score. Examples include “Driving after drinking two beers” and “Sudden braking to avoid an accident.” Items were rated on a 5-point Likert scale (1 = Not risky at all, 5 = Very Risky). Cronbach’s Alpha was 0.90 in this study, a value comparable to that reported in the original research by Rosenbloom et al. (50), where internal consistency reached 0.91 and test–retest reliability was 0.92. Similarly, in a Romanian sample, Măirean et al. (32) found a Cronbach’s Alpha of 0.88.

## 5 Data analysis

The data were analyzed using *IBM SPSS Statistics for Windows* (version 23.0; (70)), with descriptive indicators calculated and the normality assumption met. For hypothesis testing, Pearson correlation, simple and multiple linear regression, mediation and moderation analyses were computed, using *Stata Statistical Software: Release 18.0* (51).

To assess the adequacy of the sample size, a *post hoc* power analysis was conducted using G*Power 3.1 (52) for the final regression model (*F*-test, linear multiple regression, fixed model, *R^2^* deviation from zero). With an observed effect size of *f^2^* = 0.89 (corresponding to *R^2^* = 0.471), an alpha level of 0.05, 17 predictors, and a sample size of *N* = 115

The analysis revealed excellent power to detect the observed effect (1 − *β* = 0.999). This indicates that the study was sufficiently powered despite the moderate sample size.

To test indirect effects, we employed a non-parametric bootstrapping procedure with 1,000 resamples, as recommended by Preacher and Hayes (53). This approach provides bias-corrected 95% confidence intervals for the indirect effect estimates. Bootstrapping was conducted for the hypothesized mediation pathway from Illusion of Control to Risky Driving Behavior via Risk Perception. An indirect effect was considered statistically significant if the 95% confidence interval did not include zero.

## 6 Results

### 6.1 Associations between risky driving behaviors, illusion of control, desire for control, risk perception, traffic-locus of control and driving experience

The results presented in Table 1 show that risky driving behaviors correlate significantly with all other variables. Specifically, there is a small positive correlation between risky driving behaviors and the illusion of control, suggesting that individuals with a stronger illusion of control tend to engage in riskier driving behaviors. There is also a small positive correlation between risky driving behaviors and the desire for control, indicating that individuals with a higher desire for control are more likely to engage in risky driving behaviors. Finally, a large positive correlation is observed between risky driving behaviors and risk perception, suggesting that a higher risk perception is associated with an increased tendency to engage in risky driving behaviors. As levels of illusion of control, desire for control, and risk perception increase, risky driving behaviors also increase among the participants in the study.

**Table 1:** Correlations between driving experience, illusion of control, desire for control, risk perception, and risky driving behaviors overall and by factors.

Variables		D. Exp.	IoC	DoC	RPQ	TLoC 1	TLoC 2	TLoC 3	TLoC 4	TLoC 5	TLoC 6	RDB	F1	F2	F3
Driving experience		1													
Illusion of control (IoC)	*r*	0.01	1												
Desire for control (DoC)	*r*	0.06	−0.01	1											
Risk perception (RPQ)	*r*	0.17*	0.15	0.17	1										
Destiny-luck (TLoC 1)	*r*	0.10	−0.19	−0.08	0.03	1									
Religious beliefs (TLoC 2)	*r*	0.14	−0.18	−0.08	0.18	0.52**	1								
Desirability (TLoC 3)	*r*	0.16	0.22*	0.11	0.39**	−0.05	0.18	1							
Other drivers (TLoC 4)	*r*	−0.11	−0.03	0.29*	0.19*	−0.08	−0.03	0.15	1						
Internality (TLoC 5)	*r*	0.26*	0.16	0.08	0.18*	0.06	0.02	0.20*	0.16	1					
Vehicles and environment (TLoC 6)	*r*	0.27*	−0.10	0.24*	0.08	0.14	0.14	0.08	0.21*	0.26*	1				
Risky driving behaviors (RDB)	*r*	0.17*	0.24*	0.17*	0.57**	−0.19*	0.005	0.46**	0.25*	0.17	0.01	1			
(F1-RDB) Rule violations and speeding	*r*	0.22*	0.29**	0.09	0.55**	−0.15	0.03	0.48**	0.19*	0.14	−0.05	0.94**	1		
(F2-RDB) Reckless driving of others	*r*	0.08	0.03	0.22*	0.46**	−0.11	0.06	0.33**	0.27*	0.13	0.03	0.76**	0.55**	1	
(F3-RDB) Alcohol use and driving	*r*	−0.05	0.06	0.25*	0.21*	−0.25*	−0.18	0.07	0.21*	0.16	0.07	0.55**	0.29*	0.54**	1

*N* = 115; *p < 0.05; **p < 0.001; D. Exp., driving experience; TLoC, traffic-locus of control; F1,2,3-RDB, factors of risky driving behavior.

Regarding the factors, for Factor 1, attitudes toward rule violations and speeding, there is a small to moderate positive correlation with the illusion of control and a large positive correlation with risk perception. For Factor 2, attitudes toward the reckless driving of others, significant positive correlations were found with the desire for control, risk perception, attitudes toward rule violations, and attitudes toward alcohol use and driving. These correlations are positive and range from small to moderate in intensity. Factor 3, attitudes toward alcohol use and driving, correlates positively, although weakly, with the desire for control and with risk perception.

There are associations between risky driving behaviors (overall and by factors) and types of traffic-locus of control. Table 1 shows both statistically significant and non-significant correlations. For Factor 1 (attitudes toward rule violations and speeding), there is a moderate positive correlation with desirability and a small positive correlation with perceptions of other drivers’ behavior. Factor 2 (attitudes toward reckless driving of others) correlates positively with desirability and other drivers, with small correlations. Factor 3 (attitudes toward alcohol use and driving) correlates negatively with fate-luck and religious beliefs, and positively with other drivers. Overall risky driving behaviors correlate negatively with fate-luck, and positively with other drivers, internality, and Factors 1, 2, and 3. The strength of these correlations ranges from small to large.

Regarding driving experience, small positive correlations emerged with Factor 1, overall risky driving behaviors, internality, vehicle-environment traffic-locus of control, and risk perception. This suggests that more experienced drivers may better perceive traffic risks but also engage in riskier behaviors. Other correlations were statistically insignificant (Table 1). Although the study was built on the assumption that illusion of control, desire for control, and risk perception are indicators of perceived invulnerability, the results show that these variables do not significantly correlate with each other, with significance thresholds greater than 0.05 (*p* > 0.05).

In addition, the analysis of relationships between types of traffic-locus of control revealed that the illusion of control correlates negatively with fate-luck and religious beliefs, but positively with desirability, with small correlation sizes. Risk perception correlates positively with religious beliefs, desirability, other drivers’ behaviors, and internality, with correlations ranging from small to moderate. The desire for control correlates positively but weakly with perceptions of other drivers’ behavior and with vehicle-environment factors. Relationships among traffic-locus of control dimensions show that fate-luck correlates strongly and positively with religious beliefs; desirability correlates positively with religious beliefs; internality correlates positively with desirability; and vehicle-environment correlates positively with both other drivers and internality.

### 6.2 The moderated mediation model of risky driving behavior

We tested a moderated mediation model (78) in which risk perception was conceptualized as a mediator of the associations between psychological predictors (illusion of control, desire for control, and traffic-locus of control dimensions) and risky driving behavior, while driving experience was included as a moderator of the direct effects between these predictors and risky behavior. Specifically, we examined:

1. *Hypothesis*: Perceived invulnerability (i.e., illusion of control, desire for control, and risk perception) predicts risky driving behavior (H1a), with driving experience moderating these relationships (H1b).
2. *Hypothesis*: Risk perception mediates the relationships between traffic-locus of control dimensions (i.e., destiny-luck, religious beliefs, other drivers, internality, and vehicles/environment), driving experience, and risky driving behavior, even when accounting for moderation effects (H2).

#### 6.2.1 Predicting risk perception (mediator model)

**Figure 2:**
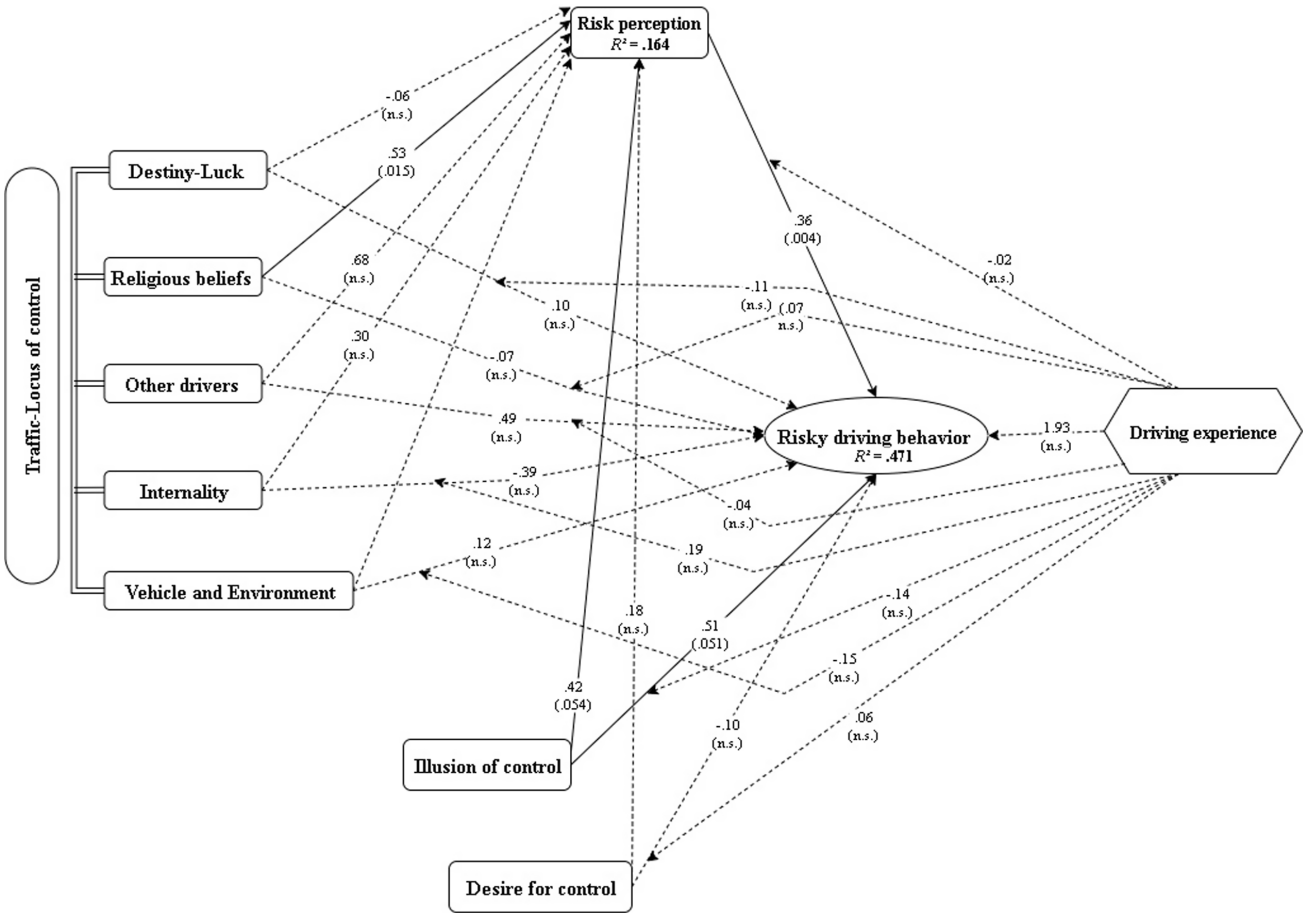
Direct and moderation effects for the tested model. n.s., nonsignificant.

To test the first stage of the model, a multiple linear regression was conducted to assess whether illusion of control, desire for control, traffic-locus of control dimensions, and driving experience predicted risk perception (RPQ) (Figure 2). The model was statistically significant, *F*(8, 106) = 2.61, *p* = 0.012, explaining 16.45% of the variance in RPQ, reflecting a small to medium effect size (54).

Among predictors, religious beliefs traffic-locus of control significantly predicted higher risk perception (*B* = 0.53, *p* = 0.015), while illusion of control showed a marginal effect (*p* = 0.054). Other predictors, including driving experience, were not significant predictors of RPQ (all *p* > 0.05). Complete coefficients are reported in Table 2.

**Table 2:** Multiple linear regression predicting risk perception (mediator model).

Predictor	*B*	SE	*t*	*p*	95% CI
Intercept	61.68	17.23	3.58	0.001	[27.53, 95.84]
Illusion of control	0.42	0.22	1.95	0.054	[−0.01, 0.86]
Desire for control	0.18	0.12	1.52	0.131	[−0.06, 0.42]
Destiny-luck (TLoC 1)	−0.06	0.15	−0.40	0.687	[−0.37, 0.24]
Religious beliefs (TLoC 2)	0.53	0.22	2.46	0.015	[0.10, 0.96]
Other drivers (TLoC 3)	0.68	0.39	1.74	0.084	[−0.09, 1.46]
Internality (TLoC 4)	0.30	0.30	0.98	0.332	[−0.31, 0.90]
Vehicles and environment (TLoC 5)	−0.22	0.44	−0.50	0.620	[−1.09, 0.65]
Driving experience	1.93	1.32	1.46	0.147	[−0.69, 4.54]

*N* = 115; *R*^2^ = 0.164, *F*(8, 106) = 2.61, *p* < 0.012, *p* < 0.05. RPQ, risk perception questionnaire; TLoC, traffic-locus of control.

#### 6.2.2 Predicting risky driving behavior

The second model tested the prediction of risky driving behavior (RDB) from all predictors, the mediator (RPQ), and interaction terms with driving experience. This combined moderated mediation model was significant, *F*(17, 97) = 5.09, *p* < 0.001, explaining 47.14% of the variance in risky driving behavior, representing a large effect size (54).

Significant predictors included: Risk perception (RPQ): *B* = 0.36, *p* = 0.004 (*β* = 0.62), indicating drivers who assess traffic situations as risky still engage in risky behaviors. Illusion of control showed a marginal effect: *B* = 0.51, *p* = 0.051.

None of the interaction terms (predictor × experience) reached statistical significance, suggesting that driving experience did not moderate the effects of psychological variables on risky driving (all *p* > 0.05; see Table 3). Although included as hypothesized, driving experience did not significantly moderate any of the tested relationships.

**Table 3:** Multiple linear regression predicting risky driving behavior.

Predictor	*B*	SE	*t*	*p*	Beta	95% CI
Intercept	−0.24	20.88	−0.01	0.991	–	[−41.67, 41.20]
RPQ	0.36	0.12	2.95	0.004	0.62	[0.12, 0.60]
Illusion of control (IoC)	0.51	0.26	1.98	0.051	0.38	[0.00, 1.03]
Desire for control (DoC)	−0.10	0.15	−0.62	0.539	−0.13	[−0.40, 0.21]
Destiny-luck (TLoC 1)	0.10	0.16	0.60	0.549	0.12	[−0.22, 0.42]
Religious beliefs (TLoC 2)	−0.07	0.24	−0.31	0.760	−0.06	[−0.54, 0.40]
Other drivers (TLoC 3)	0.49	0.41	1.20	0.233	0.21	[−0.32, 1.29]
Internality (TLoC 4)	−0.39	0.31	−1.23	0.221	−0.22	[−1.01, 0.24]
Vehicles and environment (TLoC 5)	0.12	0.51	0.24	0.813	0.05	[−0.89, 1.14]
Driving experience (experience)	5.74	8.22	0.70	0.486	0.71	[−10.57, 22.05]
RPQ × experience	−0.02	0.05	−0.52	0.605	−0.41	[−0.12, 0.07]
IoC × experience	−0.14	0.09	−1.48	0.143	−0.62	[−0.32, 0.05]
DoC × experience	0.06	0.07	0.89	0.373	0.75	[−0.07, 0.19]
Destiny-luck × experience	−0.11	0.06	−1.71	0.091	−0.55	[−0.23, 0.02]
Religious beliefs × experience	0.07	0.09	0.74	0.464	0.22	[−0.11, 0.25]
Other drivers × experience	−0.04	0.15	−0.24	0.809	−0.11	[−0.34, 0.26]
Internality × experience	0.19	0.12	1.49	0.138	0.55	[−0.06, 0.43]
Vehicles and environment × experience	−0.15	0.20	−0.75	0.457	−0.49	[−0.55, 0.25]

*N* = 115; *R*^2^ = 0.471, *F*(17, 97) = 5.09, *p* < 0.001, *p* < 0.05. RPQ, risk perception questionnaire; TLoC, traffic-locus of control.

#### 6.2.3 Indirect effects

The indirect effect of illusion of control on risky driving behavior via RPQ was tested using bootstrapping (1,000 samples). The indirect effect was not significant (*B* = 0.15, *SE* = 0.11, *z* = 1.43, *p* = 0.154), indicating that risk perception did not significantly mediate this relationship in the combined model and confirming the stability of the original findings (see Table 4). Other indirect paths were not statistically tested or significant.

**Table 4:** Indirect effect of illusion of control on risky driving behavior via risk perception.

Effect type	Estimate	SE	*z*	*p*	95% CI
Indirect effect	0.15	0.11	1.43	0.154	[−0.06, 0.36]

*N* = 115. Results based on 1,000 bootstrap samples. RPQ, risk perception questionnaire.

Overall, findings partially supported Hypothesis 1, as illusion of control and risk perception were either marginal or significant predictors of risky behavior. However, no moderating effect of driving experience was detected. Hypothesis 2 received limited support: while religious beliefs traffic-locus of control predicted higher perceived risk, the expected mediating role of risk perception in the relationship between psychological predictors and risky driving behavior was not robustly supported.

These results suggest that although psychological constructs such as illusion of control and religious attributions are associated with how drivers perceive risk, these perceptions may not translate into safer behavior. Furthermore, driving experience did not appear to buffer or amplify the influence of these variables. The mechanisms underlying risky driving remain complex and warrant further investigation using longitudinal or experimental approaches.

## 7 Discussion

The present study aimed to investigate the psychological mechanisms underlying risky driving behaviors by testing a moderated mediation model. Specifically, we examined the role of perceived invulnerability, operationalized through the illusion of control, desire for control, and risk perception, in predicting risky driving. We also tested whether risk perception acts as a mediator in the relationship between traffic-related locus of control and risky behavior, and whether driving experience moderates any of these associations. The following sections interpret these results in relation to the proposed hypotheses and existing literature.

### 7.1 Perceived invulnerability and risky driving behaviors

In the first hypothesis, we examined the role of perceived invulnerability, comprising the illusion of control, desire for control, and risk perception, in predicting risky driving behaviors. Our model explained 47% of the variance in risky driving behavior, indicating a large effect size (54) and suggesting a meaningful contribution of these psychological factors to driving risk. Risk perception emerged as a strong individual predictor, indicating that increased awareness of risks does not necessarily reduce risky behaviors and may even amplify them under certain conditions. These findings align with the research of McKenna (4) and DeJoy (49), which show that optimism bias and a false sense of safety can increase the risk of accidents. However, our results diverge from those of Baran et al. (55), Song et al. (56), and Măirean et al. (57), who found that although optimism bias is negatively associated with perceived risk, it does not necessarily translate into riskier behavior. According to these authors, risk perception may act as a suppressor, meaning that while optimistic drivers tend to downplay danger, this does not automatically lead to unsafe actions.

The observed positive association between risk perception and risky driving behavior can be understood through the lens of Risk Homeostasis Theory (42). This theory posits that individuals maintain a target level of risk and may compensate for perceived changes in safety. A heightened awareness of risk may not lead to safer actions but could, paradoxically, be a characteristic of drivers with a higher tolerance for or even a preference for a more thrilling driving experience. Such drivers may adjust their behavior to maintain a preferred level of risk, engaging in a form of risk homeostasis. Given the high rates of accidents registered in Romania (24), it is possible that Romanian drivers, as a group, are familiar with risky traffic and they have a correspondingly high acceptable level of risk. Drivers may perceive risks but believe their own skills or vehicle characteristics (e.g., ABS brakes) are superior, leading to a false sense of security that they may compensate for by driving faster or more aggressively to maintain their targeted level of risk.

Furthermore, our results align with recent research by Budak et al. (58), which found that higher risk perception is associated with more self-reported errors among drivers who attributed crashes either internally (to themselves) or externally (to others, the vehicle/environment, or fate). This suggests that for certain individuals, perceiving risk can lead to a heightened focus on personal skill, paradoxically encouraging them to take risks they believe they can overcome. The relationship between risk perception and risky driving behavior is clearly more nuanced and warrants further investigation.

The illusion of control had a marginal effect, suggesting that the tendency to overestimate one’s control over traffic events may contribute to risk-taking. This finding is consistent with previous research by Stephens and Ohtsuka (10), Holman and Havârneanu (59), and Măirean et al. (57), which highlight the role of distorted risk perception in shaping risky attitudes. However, the evidence is not strong enough to support a clear relationship.

In contrast, the desire for control was not a significant predictor, indicating that the mere need to exert control does not directly influence risky behavior in the absence of a cognitive distortion such as the illusion of control. This result contradicts some previous studies [e.g., (60)] but underlines the importance of distinguishing between the conscious desire for control and distorted beliefs about actual control. While our sample size was adequate to detect medium effects, we acknowledge that larger and more diverse samples could help clarify whether smaller effects exist. It is also possible that the relationship between desire for control and risky driving is weaker or more context-dependent in our sample, or that the current measure does not fully capture the aspects of control motivation most relevant in the Romanian driving context. Future studies could examine this construct using alternative or context-specific operationalizations to better understand its potential role in driving behavior.

Lastly, driving experience did not moderate the relationships between any of the predictors and risky behavior. This finding suggests that the psychological effects related to perceived control and risk on dangerous behavior are stable regardless of experience level. Despite its theoretical relevance, driving experience did not show significant moderation effects in our models. This suggests that its role may be weaker than expected or detectable only in larger and more diverse samples. While driving experience is widely recognized as a key factor in improving driving skills and reducing crash risk (16, 37), our results align with recent studies indicating that cognitive distortions such as overconfidence and illusion of control can persist even among experienced drivers (40, 57). In fact, recent research suggests that experience does not necessarily eliminate these distortions. For example, the illusion of control, the tendency to overestimate one’s ability to manage traffic situations, has been shown to positively predict risky driving, with risk perception acting as a mediator: drivers who overestimate their control perceive less risk and consequently engage in more hazardous behavior (57). Similarly, Navarro et al. (40) demonstrated that both novice and experienced drivers fall victim to the Dunning–Kruger effect: while novice drivers increased their confidence after simulated driving tasks, experienced drivers maintained high self-assessments regardless of actual performance. Together, these findings suggest that accumulated driving experience may improve technical skills but does not necessarily attenuate biased risk appraisal, which in turn may limit the protective effects of experience on risky behavior.

These findings are consistent with Baran et al. (55) and partially with Song et al. (56), who found that driving experience reduces risky behaviors more strongly in women than in men, women’s behaviors being fully mediated by sensation-seeking and risk perception, whereas men’s risk-taking persists due to factors such as overconfidence. However, other studies [e.g., (3, 11)] contradicted this, suggesting that driving experience plays an important role in moderating risky driving behaviors.

Moreover, the analysis of the indirect effect of the illusion of control on risky behavior through risk perception did not indicate a significant mediation. This result contradicts the hypothesis of a mediated relationship and suggests that the influence of these psychological factors manifests more directly rather than through complex causal chains.

### 7.2 Mediating role of risk perception

Regarding the second hypothesis, which proposed that risk perception mediates the relationship between dimensions of traffic-related locus of control and risky driving behaviors, the results provided only partial support. In the mediation model, only the dimension referring to religious beliefs as a source of external control significantly predicted risk perception. This suggests that individuals who attribute control over traffic outcomes to a divine or religious entity tend to perceive the driving environment as more hazardous (32, 33). However, this dimension did not directly predict risky behaviors, and risk perception did not mediate the relationship between traffic-locus of control and risky driving, as indicated by the bootstrapped indirect effect analysis.

Previous research shows that religious beliefs can influence driver behavior in different ways: in the United States, fatalistic attitudes have been associated with neglect of safety measures such as seat belt use (33), whereas in Turkey, religiosity has been linked to rule-following and lower risk-taking (34). In Romania, religious practices such as praying or placing crosses in cars often reflect a fatalistic orientation (61), which may explain why religiosity emerged as relevant for risk perception in this study [see also (32)]. These findings suggest that the role of religious beliefs is culturally specific, and future cross-cultural research is needed to examine whether similar patterns appear in societies with different religious traditions or levels of secularization.

Other dimensions of traffic-related locus of control, such as attributing responsibility to other drivers or to chance (luck/fate), were not significantly associated with either risk perception or risky behavior. These findings diverge from previous studies [e.g., (6, 10)], which reported a positive association between external traffic-locus of control and risky driving. However, they support the notion that not all forms of external control exert the same psychological influence on driving behavior.

Furthermore, driving experience did not moderate the relationships between traffic-related locus of control and risky behavior. This finding reinforces the conclusion that cognitive factors may exert a relatively autonomous influence, independent of demographic or experiential variables. Therefore, road safety interventions should focus on addressing perceptual distortions and maladaptive beliefs directly, rather than relying solely on the accumulation of practical driving experience.

## 8 Limitations

Before discussing the broader implications of the study, it is important to acknowledge several limitations that may influence the interpretation of the findings. Firstly, the study had a cross-sectional design, preventing the establishment of causal relationships between variables. Additionally, this study relied on data collected online, which may limit the generalizability of the results to the broader population of drivers. The assessment of drivers’ perceived invulnerability and risk behaviors was based on self-reports. One significant drawback of self-reported methods is the potential influence of social desirability bias, where respondents may present themselves in a more favorable light. Additionally, limited self-awareness may further affect the accuracy of the reported behaviors, making the results less reliable as indicators of actual day-to-day driving behaviors. However, given that this limitation is inherent to questionnaire-based research, future studies could focus on assessing how drivers perceive and respond to risks in real-driving contexts, possibly by using driving simulators to provide more ecologically valid data. In addition, incorporating a social desirability scale alongside self-report questionnaires would help mitigate bias and further strengthen the validity of the findings.

Although our power analysis suggests that our sample size of 115 participants is adequate for detecting medium effects in both regression and mediation analyses, we acknowledge that a larger sample would strengthen the generalizability and robustness of our findings. We recommend that future studies replicate and extend this work with larger samples. The relatively small sample size limited our ability to conduct EFA and CFA analyses to further validate our measurement model, therefore we recommend that future research with larger samples undertake such analyses to refine the model and enhance its suitability for the traffic context examined here. Similarly, due to the limited sample size, we were unable to include additional demographic variables such as age and gender as predictors in our model. Future research should explore how these demographic factors may interact with psychological variables to influence risky driving behaviors. In addition, a larger and more demographically balanced sample would allow testing additional moderators and covariates such as age and gender, which could better capture subgroup differences in cognitive biases and risky driving. Although driving experience was retained in the model as a theoretically relevant moderator, it did not show significant effects in the current analyses. Future studies with adequately powered and demographically balanced samples could incorporate these additional moderators to provide a broader understanding of how demographic factors interact with psychological predictors of risky driving.

Another essential aspect that could affect the validity of the data is the way risky driving behaviors are measured. The operationalization of this variable within the questionnaire may be considered ambiguous due to the use of the term “attitude” for individual factors and “behavior” for the overall score. This approach could be problematic, as previous research suggests that attitude and behavior do not always align consistently. Although self-reported surveys are the most widely used method in studies on drivers’ risk behaviors, future research should consider adopting more objective approaches to assess risky driving.

## 9 Implications

Despite these limitations, the study offers valuable insights into risky driving behaviors, particularly regarding the role of perceived invulnerability, conceptualized through illusion of control, desire for control, and risk perception. The findings show that these psychological factors explain a substantial portion of risky driving variance (47%), with risk perception emerging as a significant predictor. Notably, religious beliefs within the traffic-locus of control were associated with higher risk perception, but this did not translate into safer behavior. Similarly, illusion of control showed a marginal positive effect on risky driving but was not mediated by risk perception. These results suggest that while psychological constructs shape how risk is perceived, this perception alone may not suffice to reduce risky driving behaviors. Moreover, the lack of moderation by driving experience highlights that cognitive biases persist across novice and experienced drivers alike.

These findings indicate that road safety interventions must go beyond traditional skills training and directly address the distortions in judgment that sustain risky driving. For example, the illusion of control defined as the tendency to overestimate one’s ability to manage traffic situations has been linked to overconfidence and hazardous behavior. Rather than diminishing with experience, such distortions appear to persist over time (40, 57), which calls for corrective strategies. Driving simulators that provide immediate, individualized feedback have proven effective in recalibrating inflated self-efficacy beliefs, while urban design approaches such as the “shared space” model can reduce overreliance on conventional cues and encourage more attentive, less automatic driving (62, 63).

Equally important is the challenge posed by low-risk perception and unrealistic optimism. Drivers who underestimate danger or believe accidents are unlikely to affect them personally are unlikely to change behavior simply by being told that risks exist. More persuasive are emotionally engaging interventions, such as narratives from peers who have experienced accidents, which can reduce psychological distance and increase perceived vulnerability (64). Fear appeals, when paired with concrete recommendations for safe behavior, may further strengthen this reappraisal and motivate preventive action (65).

A different mechanism is evident in drivers with a strong desire for control. This motivational tendency can foster risky maneuvers, but it can also be redirected toward adaptive outcomes. Programs informed by the Health Belief Model (66) and the Theory of Planned Behavior (79) have shown that strengthening adaptive self-efficacy and building realistic confidence in one’s ability to drive safely can reduce intentions to engage in risky behaviors, particularly among adolescents and novice drivers (67).

Finally, the complex role of fatalistic and religious beliefs suggests that heightened awareness of risk is not always sufficient for behavior change. While such attributions may sensitize individuals to danger, they can also foster passivity. Interventions that emphasize social norms and personal responsibility, such as peer-based programs that encourage drivers to intervene when others act unsafely, may counteract this tendency and reinforce the belief that individual actions make a difference (24, 64, 74). In parallel, tailoring strategies to drivers’ psychological profiles, such as providing visually stimulating materials or adaptive feedback systems to sensation seekers (68, 69), may further increase the effectiveness of interventions.

Overall, these insights underscore the importance of designing road safety programs that address the specific cognitive biases and motivational patterns underlying risky behavior. By combining evidence-based psychological frameworks with innovative training methods and cultural sensitivity, interventions can more effectively challenge maladaptive beliefs, recalibrate distorted risk perceptions, and ultimately promote safer driving across all levels of experience.

## 10 Conclusion

This study highlights the significant role of psychological factors, particularly risk perception and illusion of control, in predicting risky driving behavior. Driving experience did not moderate these effects, suggesting cognitive biases influence drivers across all experience levels. The nuanced impact of traffic-locus of control dimensions indicates the need for tailored interventions targeting maladaptive beliefs. Future research should employ longitudinal or experimental designs to further clarify these complex relationships and support effective road safety strategies.

## Data Availability

The raw data supporting the conclusions of this article will be made available by the authors, without undue reservation.
